# The metaverse as opportunity for architecture and society: design drivers, core competencies

**DOI:** 10.1007/s44223-022-00010-z

**Published:** 2022-08-17

**Authors:** Patrik Schumacher

**Affiliations:** 1 Zaha hadid architects, London, UK; 2 Architectural Association School of Architecture, London, UK

**Keywords:** Metaverse, Cyberspace, Phenomenology, Semiology, Dramaturgy, Liberland

## Abstract

The thesis is that the metaverse will become a pervasive part of the future internet and will thus become a key arena within which the life of society unfolds. As three-dimensional, immersive virtual world, the metaverse will be designed by architects rather than graphic designers. After 30 years of theoretical speculation and technological advances the internet is finally on the way to transforming in ways envisioned with the concept of ‘cyberspace’. The key analogy is no longer the magazine with separate pages but the city and its seamless web of spaces. The paper argues that this immersive internet delivers a superior, more productive platform for social exchange and communication. Co-location synergies will unfold and order the distribution of sites and enable an intuitive browsing navigation full of discoveries and serendipitous encounters, as well as creating sites for vivid crowd interactions. It is this superiority that will lead to architects taking over from graphic designers as profession owning the design of all online interaction frames. This paper explores the plausibility of this takeover and the attendant expansion of architecture’s competency.

## Introduction

The life process of society is a communication process that is ordered via a rich typology of communicative situations. It is the designed environment, both physical and digital, that spatially distributes, frames, stabilises and coordinates these distinct situations within an evolving order that allows us to self-sort as participants of various specific social interactions. Designing is communicative framing. This task formula and understanding of the societal function of architecture and design applies also to web design and the framing of all digitally mediated forms of social interaction. This insight must now be made the explicit premise and agenda for a systematic design research project that bridges architecture and interaction design in 3D virtual worlds. All the design disciplines, from urban design and architecture to fashion and graphic design, together do or should form a unified discourse and practice with a unity of purpose: the sensuous framing of communicative social interaction. This also includes all web design, all video-conferencing platforms, as well as all virtual collaboration platforms. Here too our colleagues’ framing design work is always involved.

A metaverse is an ensemble of virtual environments in which a new form of communication via personal avatars can happen across global computer networks like the world wide web. Metaverse spaces are 3D or 4D digital models, augmented with interaction capabilities. They are accessible from devices like personal PCs, laptops, mobile apps, smart TVs and VR headsets, as well as – in the near future – from architectural spaces that double up as interfaces via panoramic screens, or holographic projections. Massively multiplayer online (MMO) video-game technologies, combined with high-speed network and cloud technologies, enable such virtual spaces to be immersive, sensorially engaging platforms of information sharing and real time communicative interaction. Such interactions include social networking, creative collaboration, conferencing, exhibitions, public performances, and many types of commercial interactions. The metaverse is thus enabling novel markets and novel social, cultural, educational and business exchanges.

The metaverse is being built as we speak, rapidly. But who is designing it? Who should design it? The thesis here is that the design of the metaverse falls within the remit and core competency of the discipline of architecture.

## Architecture’s core competency

Architectural design for cyberspace—while implying a total substitution of the entire engineering stack—follows the same premises that guide architectural design in general. This expansion of architecture’s remit will further distil the discipline’s essence and core competency, namely the spatio-visual ordering of communicative interaction, upgraded via investment into the design drivers and sub-disciplines of spatiology, phenomenology, and semiology. Matters of communication and communicative framing, dynamic or static, are here – in the context of metaverse design—distilled as the essential function of all design. The native interactivity and malleability of the digital realm suggests ‘dramaturgy’ as yet another sub-discipline and key ingredient of our discipline’s competency. In the context of the design of virtual communication spaces it makes sense to interpret the architect’s task as UI/UX design. This concept of an interface design and user experience design can be generalized across all design disciplines, including architectural design in its physical instantiation. All architecture and design is UX/UI design, involving organisation (spatiology), articulation (phenomenology), signification (semiology) and interaction design (dramaturgy).

In the realm of virtual environment design the desire for adaptive, kinetic, actively self-transforming communicative architectural frames is much easier to realise. On this count dramaturgy will be much more foregrounded in the metaverse than it ever was in the context of physical architecture.

There are three premises of metaverse design posited here and implied by the author’s theory of architecture (Schumacher, 2010): a general premise concerning the scope of the design disciplines, a general premise about the societal responsibility of design, and a premise concerning the specific task posed by the current societal condition:

### Premise 1: the universal power of design

Architecture and the design disciplines together bear universal and exclusive responsiblity with respect to the phenomenal appearance of the totality of the build environment and the world of artefacts, i.e. with respect to the totality of our phenomenal world. Appearances are important contributors to social functionality: they are indispensible orienting communications. Everything that surrounds us, meets our senses and, as interface, mediates our communications with the social world was visualized by a colleague designer. The design disciplines include: urban design, landscape design, architecture, interior design, furniture design, fashion design, product design, as well as graphic design and web-design, and in the near future: metaverse design.

### Premise 2: framing as societal responsibility of design

The societal function of all design – physical or virtual – is the spatio-visual framing of communicative interaction. The designed spaces – physical or virtual—are themselves communications: they are communications that define, premise and prime the communicative interactions that are expected to take place within the respectively framed territory. These framing communications are crucial aspect in ordering society by differentiating, defining and structuring specific social situations with specific purposes, social protocols, and conditions of success.

### Premise 3: the task posed by the contemporary condition

With respect to our complex and dynamic ‘postfordist network society’ the design task of framing implies now: Complex, dynamic, dense, and diverse (but legible) information-rich environments are to facilitate orientation, navigation, and recognition (the identification of specific social situations) for an interaction-rich, productive, communicative, collaborative social life process. This applies equally to the design of physical and virtual environments or frames.

All the design disciplines together form a single discourse and function system of society with the shared societal function of the spatio-visual framing of all communicative interactions. This discourse revolves around the lead distinction of form (internal reference) versus function (external reference), is structured by the persistent binary code of (formally and functionally) ‘resolved’ versus ‘unresolved’. The criteria for the concrete application of these code values is being programmed and re-programmed by the design disciplines’ historically evolving, adapting *styles*. To the extent that all designers respond to the global design discourse, this discourse is omnipotent with respect to the spatio-visual shaping of the phenomenal world, within the economic constraints and social functionality expectations set by (private or public) clients.

The design task of the communicative spatio-visual framing of the societal life process must draw on four distinct but interrelated domains of expertise with their respective subtasks as follows:

- spatiology: organisation delivering complex configurations
- phenomenology: articulation delivering perceptual tractability
- semiology: signification delivering information-richness
- dramaturgy: interaction delivering interaction-richness

These four domains of learning define four agendas and parts of every architectural design project: The spatiological project, the phenomenological project, the semiological project and the dramaturgical project. Together these four parts constitute the methodological structure of the design project and process, for the design of both physical and virtual environments. These four subtasks and domains of expertise can be further defined and characterized as follows:

‘Spatiology’ is concerned with spatial organisation and plots out the geometric premises, sets the scene as it were, for the other three agendas of phenomenology, semiology and dramaturgy. Spatiology guides the distribution of places in space with respect to distancing, adjacencies and connections. This task dimension involves the selection of strategies of spatial organisation like axial ordering, grids, stacking, nesting, overlapping etc. An expanded spatiological expertise might draw on conceptual and computational resources provided by the mathematics of network theory.

‘Phenomenology’, as understood in this essay (and in the author’s theory of architecture in general), is concerned with morphological articulation and addresses the problem of the perceptual tractability of complex spatial/social scenes, i.e. the task of maintaining legibility in the face of complexity (Schumacher, 2012). The psychology of perception in general, and the subfield of spatial cognition in particular, provides a key resource with respect to this task dimension.

‘Semiology’ is concerned with communication via signification. The meaning of spaces and designs coincides with the social interactions they frame, i.e. meaning is or anticipates social use (Schumacher, 2012). The semiological project sets out the task of increasing the information-richness of the built environment by means of crafting a spatio-visual language or system of signification that is empowered by the combinatorial potency of grammar. Here linguistics serves as a fertile source domain for conceptual inspiration.

‘Dramaturgy’ is concerned with patterns and potentials of interaction. In the context of architecture, and in the context of designing for the metaverse, understood as a spatial immersive world wide web, dramaturgy is closely related to what in web-design is pursued under the heading of interaction design. Dramaturgy implies environmental action in the time dimension, i.e. the design of a built environment, physical or virtual, that is both kinetically responsive to user interaction as well as spontaneously engaging users (Schumacher, 2021).

Spatiology, phenomenology, semiology and dramaturgy identify and define central aspects of architecture’s task domain. These aspects of architectural learning respond to key challenges contemporary architecture faces with respect to its need to adapt its intelligence and design resources to the density, complexity and dynamism of twenty-first century social life. It is especially in this context of cyberspace and metaverse design that the task dimensions of spatiology, phenomenology, semiology and dramaturgy as key dimensions of a spatial UI/UX design effort are foregrounded as indispensable competencies. Here too architecture must cope with increasing levels of social communicative density, complexity and dynamism. As physical construction constraints disappear, spatial cognition, information density and interaction richness come to the fore as the critical performance criteria of design.

## The overdue advent of cyberspace and metaverse

The internet started as a mainly academic network in the 1980s and took off more broadly in the early 1990s. Soon some of us architects imagined that the internet would develop into a virtual three-dimensional navigation and communication space, i.e. ‘cyberspace’. The word “cyberspace” was coined by science fiction writer William Gibson, in his 1984 science-fiction novel ‘Neuromancer’. The term ‘metaverse’ was coined by Neal Stephenson in his 1992 science-fiction novel ‘Snow Crash’.

The design studio the author was teaching at TU Berlin in 1995 was exploring this idea under the heading ‘Virtual College’: Online learning as collective experience facilitated within a virtual architecture. Informative inspiration was drawn from architect Michael Benedikt’s seminal book, first published in 1991: ‘Cyberspace: First Steps’. Benedikt mused about “a new stage, a new and irresistible development in the elaboration of human culture” (Benedikt, 1991a, p.1) and did speculate conscientiously and resourcefully about “the nature of the artificial or illusory space(s) of computer-sustained virtual worlds” (Benedikt, 1991b, p.119).

However, the internet became a magazine-like medium instead, the preserve of graphic designers rather than architects. This will change. Cyberspace and metaverse are now firmly on the agenda.

Due to the long drawn out Covid-19 lockdown experienced across the world in 2020, 2021 and into 2022, all communication, work collaboration, and all social events were pushed online, into the realm of digitally mediated interaction. The adoption of video-conferencing tools shot up massively, and so did the business investment into this domain. We are currently witnessing an explosion of start-up companies offering virtual event spaces. This new situation accelerated a process that had been going on for a while. But mass adoption brings a wholly new dynamic into this realm.

This re-emergence of the idea of cyberspace, this time with accelerating practical pressure and much more technological power than 30 years ago, was rather sudden. Michael Benedikt’s book remains a valid resource of inspiration.

Benedikt asks (and gives answers to) the key questions that remain relevant: “How might it (cyberspace) look like, how might we get around in it, and, most importantly, what might we usefully do there?”(Benedikt, 1991a, p.19). The last of these most general questions should probably be answered like this: We would want to do there everything we are doing in urban and architectural spaces: browse, communicate, work, learn, create, both individually and collaboratively, socialise, entertain etc. The lockdown has impaired all urban and architectural interaction spaces and thus calls for everything to go virtual. This is a radically new situation. In the intervening years virtual environments were a choice, not a necessity, and the choice in favour of VR was made primarily in the realm of entertainment, especially via video games. This market had grown sufficiently large to deliver resources to technological development, ample user market feedback, and a whole competitive industry. The fruits of these investments can now be reaped via technology transfer into societal domains where serious productive work is to be facilitated for adult users who have no time to waste. The forced push due to Covid-19 has led to the discovery that remote, mediated collaboration can be effective. This lesson cannot be un-learned and a new working lifestyle starts to emerge and stay. The thesis of this paper is that this new life will be based on cyber-urban integration and metaverse agglomeration.

Benedikt asks further: “Which axioms and laws of nature ought to be retained in cyberspace … and which axioms and laws can be adjusted or jettisoned for the sake of empowerment.” (Benedikt, 1991b, p. 119). This is an important question, and there are many possible answers. In any event, cyberspace will have a “geography, a physics, a nature, and a rule of human law” (Benedikt, 1991b, p.123). Benedikt shares some useful considerations and proposes some heuristics he discovered in the speculative cyber-space design explorations he conducted with his students. He rightly suggests that when cyberspace takes off “there will likely be myriad places in, and many regions of cyberspace – each with its own character, rules and function” (Benedikt, 1991b, p.122). He also anticipates that there will be a number of different competing kinds of cyberspaces, “each with its own culture, appearance, lore and law” (Benedikt, 1991b, p.122).

Benedikt introduces some useful basic distinction, like the distinction ‘navigation versus destination’, and the distinction ‘extrinsic versus intrinsic’ dimensions (Benedikt, 1991b, pp.134–135). These are dimensions of information encoding or visualisation, whereby the extrinsic dimensions are the two or three spatial dimensions that define an object’s location or position in space (with time being a fourth extrinsic dimension) while an unbounded number of morphological properties or features are brought under the notion of intrinsic dimensions that might be used to distinguish and characterise an object or place in cyberspace. The important insight is put forward here that, with respect to the function of information conveyance, extrinsic and intrinsic encodings are in principle functionally equivalent, so that it is the cyberspace-designer’s choice which aspect or information to encode via extrinsic variables, i.e. (absolute or relative) location/position, and which via intrinsic variables, i.e. shape, colour, materiality etc. The presumption here is—just as in the case of an urban order—that spatial positions are not randomly allocated but mean something and thus convey some (at least probabilistic) information.

While Benedikt does not reference architectural semiology, probably because he conceives cyberspace more in terms of data-visualisation than in terms of architecture and spaces of interaction, it became clear to the author in the early 1990s that cyberspace design is essentially an effort in architectural semiology. However, while the engagement with cyberspace was left behind (because the web became instead the domain of graphic designers) but the author’s keen interest in the semiological project as a central aspect of the architect’s core competency remained. With this came the theme of ‘information-richness’, or ‘information density’ in the author’s theory of architecture (Schumacher, 2013), a theme which was also one of Benedikt’s central themes for cyberspace design. The other theme that was brought back into architecture and urban design is the theme of orientation and navigation. Now the renewed engagement with the problem and task of cyberspace design brings these central themes back full circle. Architectural theory is thus well prepared for the challenge of metaverse design.

The distinction of navigation and destination is not a strict one. Most urban and architectural spaces are both navigation and destination spaces. The differentiation of pure navigation spaces like corridors, highways and subways are a modern phenomenon, but even these spaces are never wholly devoid of information and communication potentials but can offer more than the mere transition from A to B. The city can and should be browsed, and this browsing should also be a keen mode of engagement with cyberspace. We cannot assume that users know about all the offerings in advance but rather they must be enabled to browse, scan and discover what is there, not utterly randomly but in a structured exploration, where serendipitous discovery is enabled without a loss of overall orientation. Virtual environment researchers Rudolph P. Darken and Barry Peterson make this point too:

Navigation is rarely, if ever, the primary task. It just tends to get in the way of what you really want to do. Our goal is to make the execution of navigation tasks as transparent and trivial as possible, but not to preclude the elements of exploration and discovery. Disoriented people are anxious, uncomfortable, and generally unhappy. If these conditions can be avoided, exploration and discovery can take place. (Darken & Peterson, [2018] p.468)

The explorative surplus navigation can bring, as an alternative to just tele-porting instantly to pre-selected destinations, has its equivalent in the slackness of lingering time around scheduled events. Informal pre-gatherings and the post-event lingering are very important for networking and an informal information exchange. These spontaneous networking processes make productive use of the non-random, select group brought together by the respective scheduled event, e.g. by a lecture, conference or exhibition opening etc. The utilisation of such an opportunity for explorative meetings and information exchanges requires structured spaces of extended co-presence that are not available via conferencing tools like zoom, or in virtual exhibitions, both still based on the magazine or page analogy rather than the city and building analogy.

To return to Benedikt’s question which axioms and laws of nature ought to be retained in cyberspace: The same question is posed with respect to the familiar organisation and articulation of the city, its buildings and spaces. How much of this must be retained in order to effectively exploit the city analogy? The point of the analogy is to build on our familiarity with cities and on our collectively shared competency as city dwellers and users. This knowledge is extensive, and mostly tacit, and therefore underestimated. The panoply of building types and types of urban or interior spaces that order our interactions in real space is highly differentiated, full of nuanced clues about the social situations and protocols of communication competent participants have to distinguish and master. The ‘laws of the city’ are much richer than the laws of nature. They are not universal a priori constraints but have co-evolved together with the societies they sustained, and must be understood historically, as embodying a historically transient pragmatic rationality. Due to the fact that the expressed information is mostly taken in subconsciously, and not easily made explicit, a strategy of close analogy and realistic rendering, rather than radical abstraction and other-worldliness, recommends itself as initial default strategy. This does not imply the wholesale replication of familiar spaces. Semiological associations can also we carried forward, intuitively engaged and activated with original creative designs, as long as they are grounded in the base vocabulary and grammar of the contemporary city. Although there is an inherent tension between the demands for novelty and familiarity, avant-garde styles like tectonism – the latest stage of parametricism—can be employed to great effect as long as a deliberate semiological project is pursuit.

## Metaverse design innovations feeding back into physical architecture

While Benedikt presciently predicted the currently emerging virtual worlds and meta-verses when he talked about cyberspace as “a new universe, a parallel universe created and sustained by the world’s computers and communication lines” (Benedikt, 1991a, p.1), the emphasis here is instead on the integration and indeed fusion of physical and virtual spaces.

When tasked with the simultaneous design of both the physical and virtual environments for a client the question also becomes: To which degree will the virtual extensions of the physical architecture retain its look, feel and logic? Probably to a very large extent, especially if we allow the new design features motivated by the modus operandi of the virtual expansion to feed back into the design of the spaces of physical co-presence. Even if the dramaturgy is different, the semiological system of signification should be largely the same and cross the divide between physical and virtual realms.

This feedback or influence of the virtual design into the physical design should include attempts to physically implement the pro-active, adaptive, mobile architectural agents we can presume to be pioneered more pervasively in the virtual domain. The virtual domains will also effortlessly advance additional (real time) graphic information overlays. These too should, as much as possible, be implemented in the design of the physical interaction domains, via AR devices like Google/Facebook glasses, via projections, or if no real time variability but only static information is applied, via further permanent morphological or material encoding. The presumption (and promoted heuristics) here is the massive increase in information density, both in the virtual and in the physical spaces, far beyond what we are used to encounter in architecture and urban design up to now. The hypothesis and hope in this respect is that the advent of cyberspace will lead to a new flourishing of architectural semiology. This is plausible or can be expected to the extent to which cyberspace will, from the perspective of its users, surpass any known city in terms of its variety and density of differentiated, effective interaction offerings. For this density to remain navigable, semiological (as well as phenomenological) articulation will become necessary. Large proprietary complexes or districts will probably be semiologically integrated by their dedicated or coordinated designers while larger city-like agglomerations will engender a spontaneous semiosis that then feeds on itself in its further expansion and densification. In any event, architectural semiology, as the (still largely unacknowledged) essence of the metaverse design task, has a better chance to succeed in cyberspace than in physical urban space, not least due to the fierce global, borderless competition in cyberspace, and due to the attendant more rapid historical turnover and remodelling of spaces. The increased communicative capacity that will then increasingly be expected by the users of cyberspace will lead them to expect or demand a similar information richness and communicative capacity from the physical urban and architectural spaces they would be willing to patronise. The users’ expectations and the competency in information absorption they acquired in cyberspace will fuel and finally force the semiological upgrading of the physical environment too. The challenge will be the faster pace of development, continuous upgrading, replacement. We have to get used to that we are not building for eternity.

This physical environment will not only acquire a new semiological density and coherence but will be transformed in many further respects as it gets enveloped by and infused with virtuality. Most walls and architectural and urban surfaces will become windows into virtual extensions connecting physical to virtual spaces. Room-sized, full or partially enveloping panoramic screens or projections are very effective mechanisms of collective immersion into virtual spaces. Whole groups of physically co-located participants can thereby be transported into a virtual environment, and thereby interact with several other groups. Another potent form of tele-presencing is the use of holograms. The required equipment could be built into strategic locations like at the lectern in a lecture theatre. Both technologies are being advanced rapidly, to ever greater effect and are ever more affordable. A further compelling technology for tele-presencing is Microsoft’s VROOM—Virtual Robot Overlay for Online Meetings. Here telepresence robots allow remote users to freely explore a space they are not in, and provide a physical embodiment in that space. Here a robot acts on behalf of a remote participant in a physical space, just like an avatar in a virtual space. That robot is either equipped with a screen at head height to deliver a video presence of the remote participant, or becomes the site of an AR overlay for co-present participants wearing AR glasses. Holograms might also be spawned from such robots. These examples in hardware evolution imply that we must not imagine that cyberspace will be experienced only at home from a laptop, phone or headset, but within new types of technology empowered immersive spaces.

## Cyber-urban incubators

Recent urbanism notes and facilitates industry-specific urban knowledge economy clusters delivering valuable co-location synergies. Within the individual buildings the synergetic clustering of mutually relevant firms and their spaces is repeated. The idea on a Cyber-Urban Incubator is proposing to double up these urban spaces with corresponding virtual spaces, not as digital twin replicas but as congenial extensions with their own related laws of navigation, encounter and modes of interaction. However, these two realms should ideally be connected via spatial interfaces and via a unified spatio-visual language as integrated system of signification. Physical spaces can afford windows into virtual spaces where the semiology is the same and the logic of gathering and communicating is similar enough to allow for the transfer of competencies from the physical to the virtual realm. These competencies are the complementary competencies of both designers and end users.

The project presented below to illustrate the idea of cyber-physical hybridity is a post-graduate design research thesis developed in the context of the Design Research Lab at the Architectural Association (AADRL) in London. The project, developed by a team of four graduate students, was part of cluster of four such projects forming a creative industry start-up urban district at the Queens waterfront in New York.

**Fig. 1:**
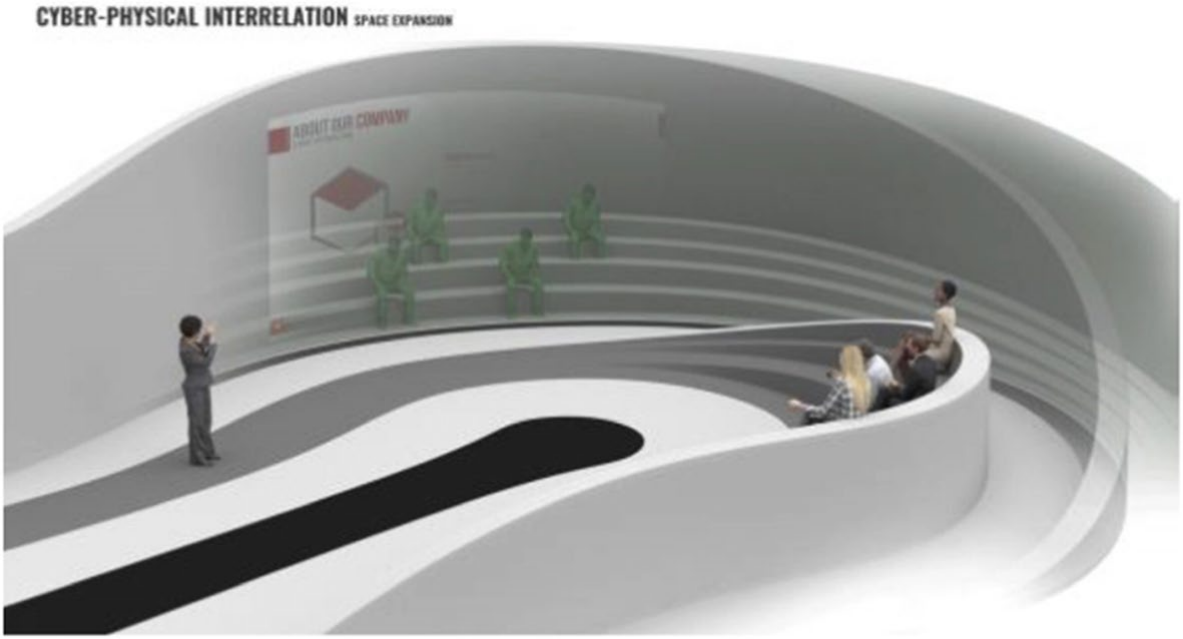
Trompe-l'œil space expansions. ‘Cybertecture’ by Stefan Tzon Manousof, Qi Yang, Amin Yassin, Yang Yu; AADRL Studio ‘Cyber-urban Incubator’, tutors: Patrik Schumacher, Pierandrea Angius

**Fig. 2:**
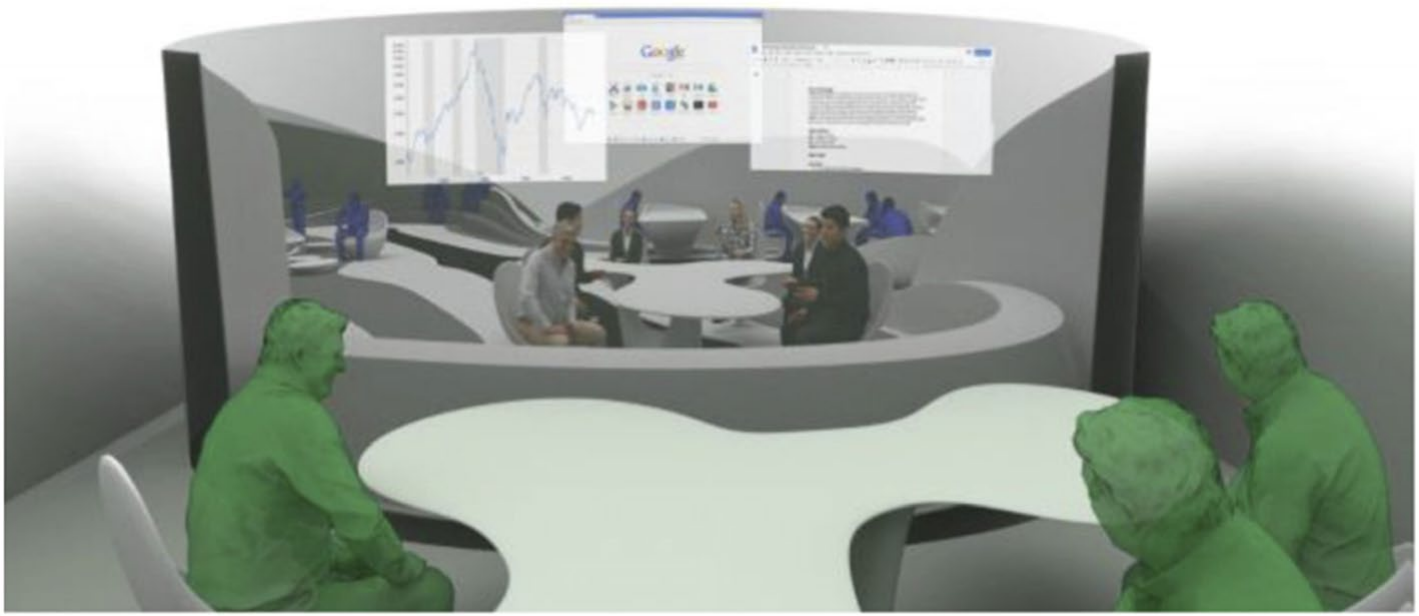
Trompe-l'œil space expansions. ‘Cybertecture’ by Stefan Tzon Manousof, Qi Yang, Amin Yassin, Yang Yu; AADRL Studio ‘Cyber-urban Incubator’, tutors: Patrik Schumacher, Pierandrea Angius

**Fig. 3:**
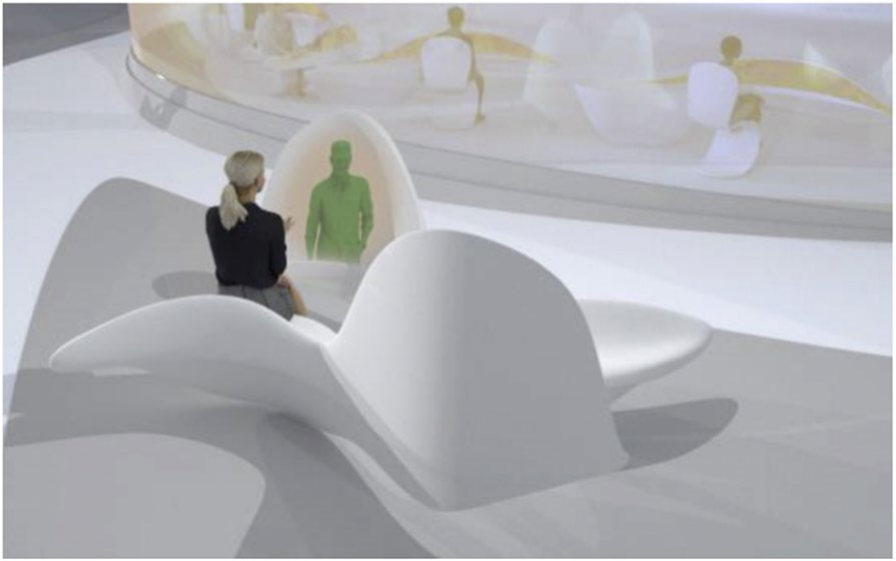
Trompe-l'œil space expansions. ‘Cybertecture’ by Stefan Tzon Manousof, Qi Yang, Amin Yassin, Yang Yu; AADRL Studio ‘Cyber-urban Incubator’, tutors: Patrik Schumacher, Pierandrea Angius

**Fig. 4:**
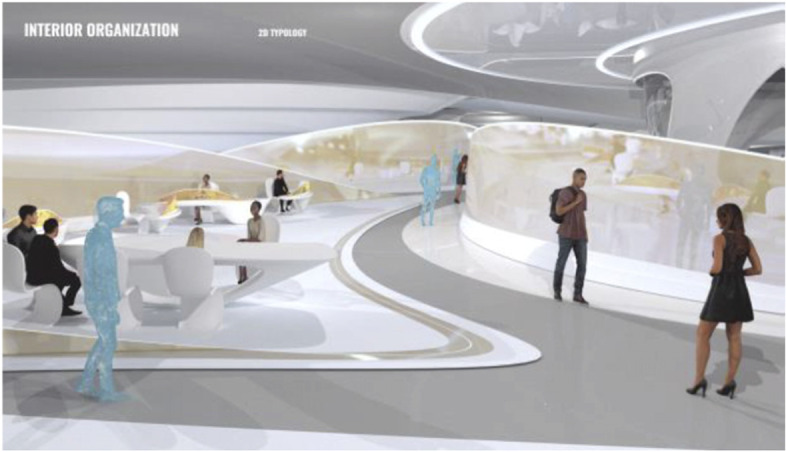
Trompe-l'œil space expansions. ‘Cybertecture’ by Stefan Tzon Manousof, Qi Yang, Amin Yassin, Yang Yu; AADRL Studio ‘Cyber-urban Incubator’, tutors: Patrik Schumacher, Pierandrea Angius

The project started by conceptualising and sketching a series of hybrid interaction scenarios. There are a number of technologies that allow for the tele-presence of remote participants, for instance holographic projections, or more advanced options like VROOM, Microsoft’s tele-presence option via remote controlled robots. Low tech options for tele-presence include the possibility of mixing physical presence with remote participation by interweaving physical chairs with virtual chairs (screens) around a table. Another intriguing option is to work with large panoramic screens that function as a virtual expansion of the physical space, similar to a trompe-l'œil, but inhabited by avatars representing the remote participants (Figs.1, 2, 3 and 4).

**Fig. 5:**
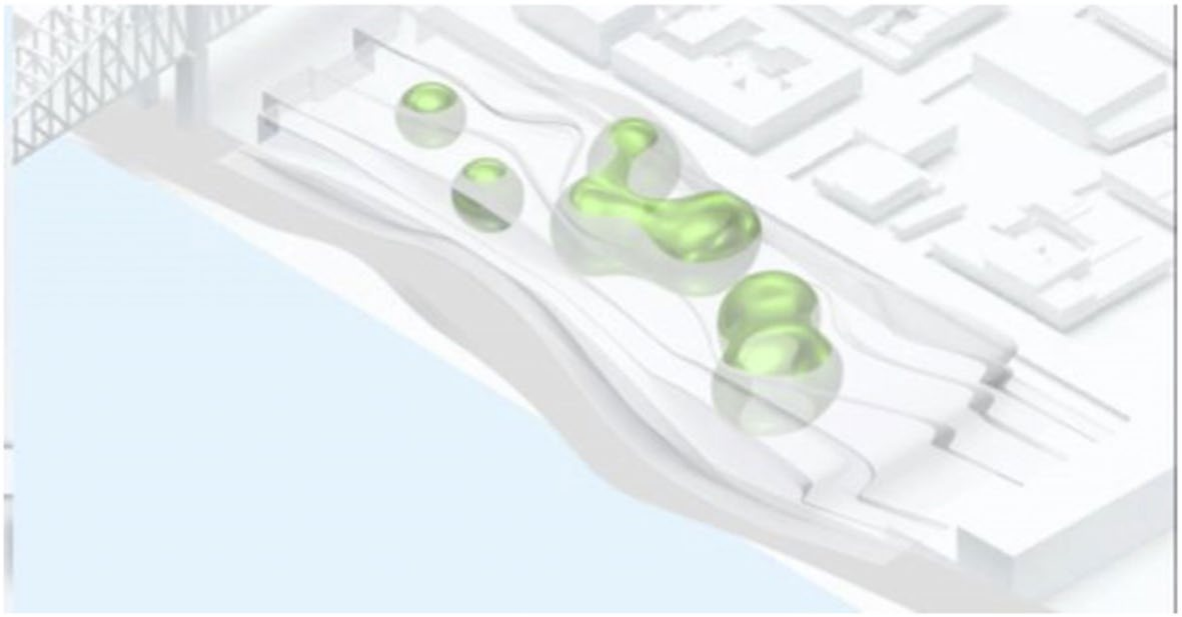
Spatiology and phenomenology of the virtuality voids. ‘Cybertecture’ by Stefan Tzon Manousof, Qi Yang, Amin Yassin, Yang Yu; AADRL Studio ‘Cyber-urban Incubator’, tutors: Patrik Schumacher, Pierandrea Angius

**Fig. 6:**
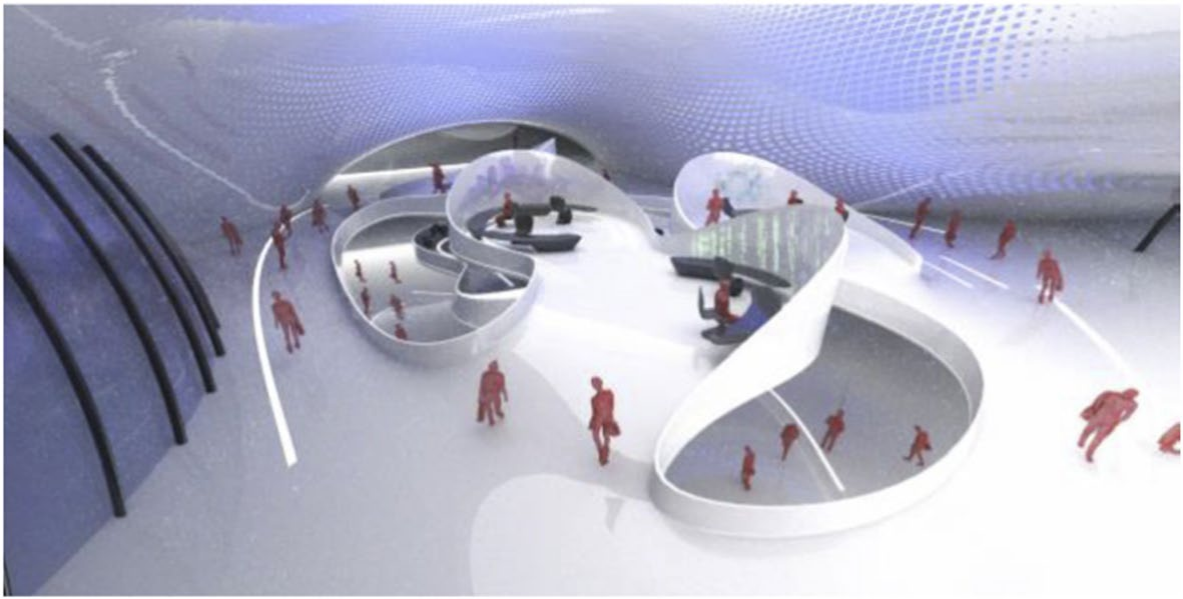
Spatiology and phenomenology of the virtuality voids. ‘Cybertecture’ by Stefan Tzon Manousof, Qi Yang, Amin Yassin, Yang Yu; AADRL Studio ‘Cyber-urban Incubator’, tutors: Patrik Schumacher, Pierandrea Angius

**Fig. 7:**
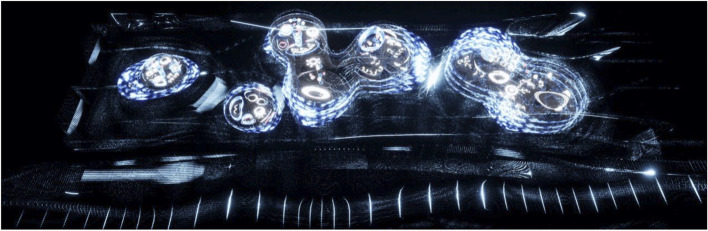
Spatiology and phenomenology of the virtuality voids. ‘Cybertecture’ by Stefan Tzon Manousof, Qi Yang, Amin Yassin, Yang Yu; AADRL Studio ‘Cyber-urban Incubator’, tutors: Patrik Schumacher, Pierandrea Angius

**Fig. 8:**
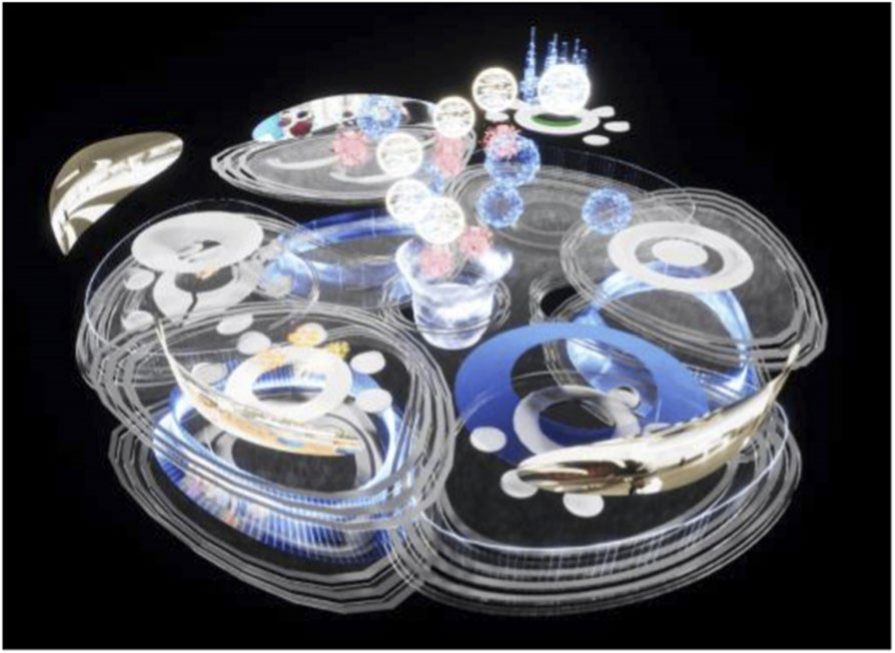
Spatiology and phenomenology of the virtuality voids. ‘Cybertecture’ by Stefan Tzon Manousof, Qi Yang, Amin Yassin, Yang Yu; AADRL Studio ‘Cyber-urban Incubator’, tutors: Patrik Schumacher, Pierandrea Angius

**Fig. 9:**
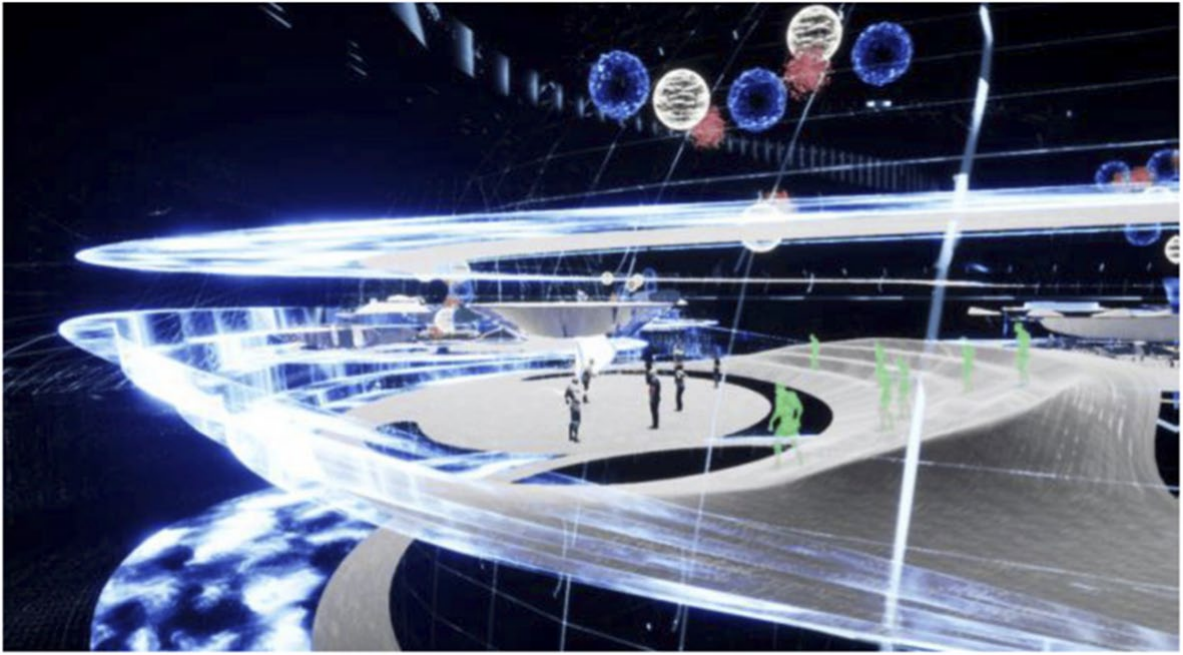
Spatiology and phenomenology of the virtuality voids. ‘Cybertecture’ by Stefan Tzon Manousof, Qi Yang, Amin Yassin, Yang Yu; AADRL Studio ‘Cyber-urban Incubator’, tutors: Patrik Schumacher, Pierandrea Angius

These projected space expansions function like windows into virtual spaces. These virtual spaces continue the geometry of the physical space and simulate its materiality via photorealistic real time rendering as is now possible, for instance, with the latest version of Epic’s ‘unreal engine’. However, geometric and stylistic continuity at the edges might be enough to establish a sense of immersive connection with the physical space, allowing then also for inventive transformations of the virtual space expansion, like various degrees of abstraction, or an enhanced kinetic malleability. A further obvious possibility that is being explored in the AADRL studio is the opportunity to work with info-graphic overlays that deliver further information about the space and its participants. These overlays can also be overlaid onto the physical space via augmented reality (AR) technology. The design inspiration flows in both directions of the physical – virtual divide (Figs. 5, 6, 7, 8 and 9).

The spatiological strategy for the project assumes that the intensity with which physical spaces are laced with connections into virtual realms is a matter of degree. There are areas where physical presence dominates, like in current office environment where only the desk tops, laptops and mobile phone screens connect with the virtual world, without giving a sense of spatial integration. On the other end of this spectrum we find spaces, like VR caves, where the immersion into virtual interaction scenarios dominates, even when several individuals are also physically co-located while joining a virtual space. The spatiology here distributes a series of blob-shaped voids that cut across the building’s slabs to establish the places with a high degree of virtuality as multi-storey mega-caves that are also visible on the exterior of the building as glowing, animated domes. These mega-blobs occasionally also form meta-ball formations. Between these high virtuality centres and the low virtuality periphery users can follow a gradient of variously calibrated mixed reality spaces that are ordered in a trajectory of increasing mediation towards the virtuality nodes.

In terms of phenomenology and semiology, the gradient is reinforced in the following way: Those local space expansions that are closer to the periphery and where physical co-location is foregrounded and dominates the mixed reality scenario, the virtual spaces are rendered in photo-realistic fashion, mimicking closely the form and materiality of the physical space. As the mixed reality spaces approach the voids, and the balance between physical and virtual presence shifts, the way the virtual spaces are rendered also shifts towards abstraction, increased kinetic malleability and generally towards greater and greater emancipation from the presuppositions of the physical design. The degree of rendering abstraction and design emancipation displayed in the virtual space expansions is thus signifying the degree to which the respective communication events are locally or globally focussed. The opportunity and intensity of dramaturgical design ideas also increases with the increasing shift of balance towards the virtual realm. However, dramaturgy is never altogether absent even from physically focused spaces.

**Fig. 10:**
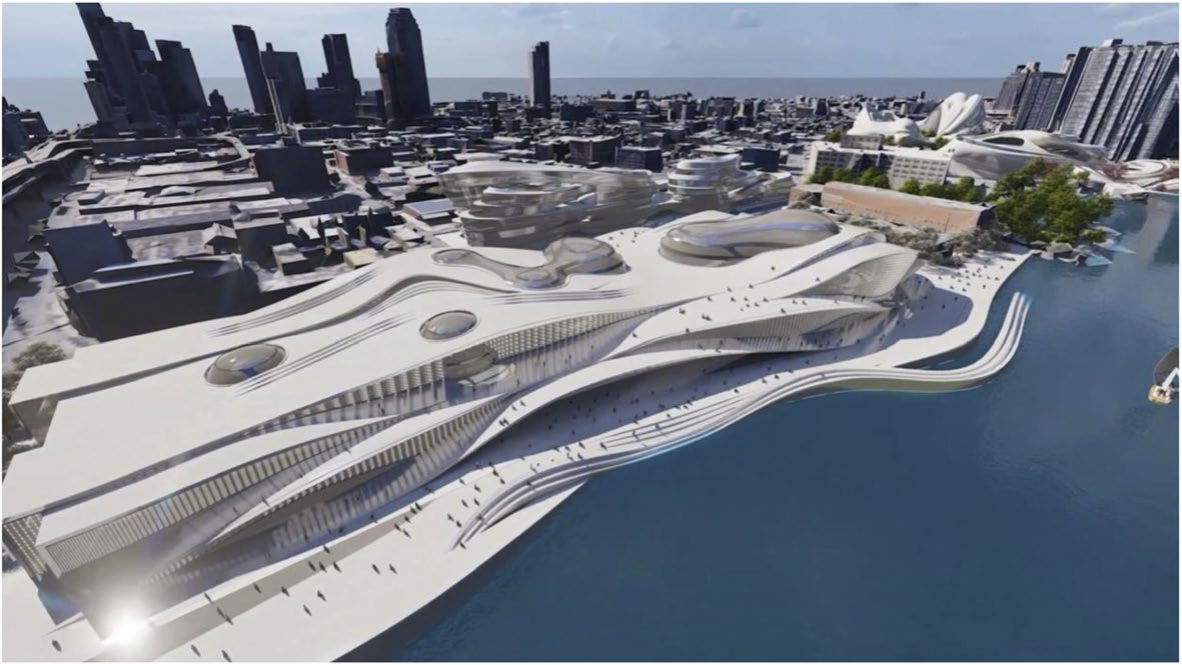
Urban interface of the cyber-urban incubator – façade and courtyard. ‘Cybertecture’ by Stefan Tzon Manousof, Qi Yang, Amin Yassin, Yang Yu; AADRL Studio ‘Cyber-urban Incubator’, tutors: Patrik Schumacher, Pierandrea Angius

**Fig. 11:**
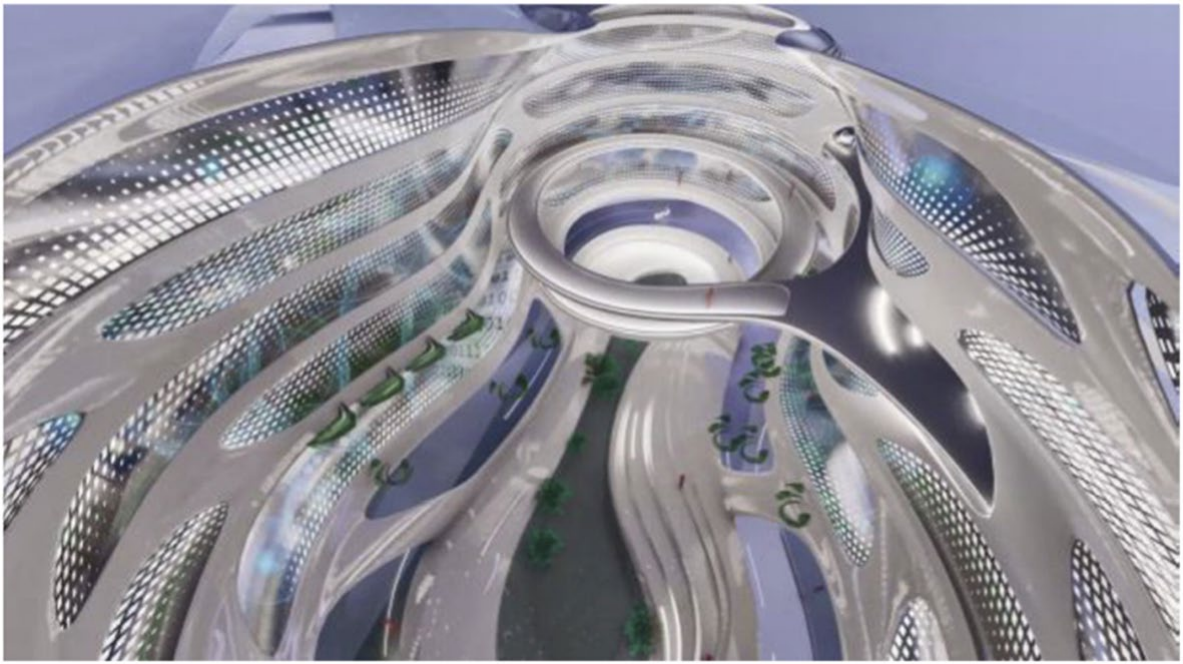
Urban interface of the cyber-urban incubator – façade and courtyard. ‘Cybertecture’ by Stefan Tzon Manousof, Qi Yang, Amin Yassin, Yang Yu; AADRL Studio ‘Cyber-urban Incubator’, tutors: Patrik Schumacher, Pierandrea Angius

**Fig. 12:**
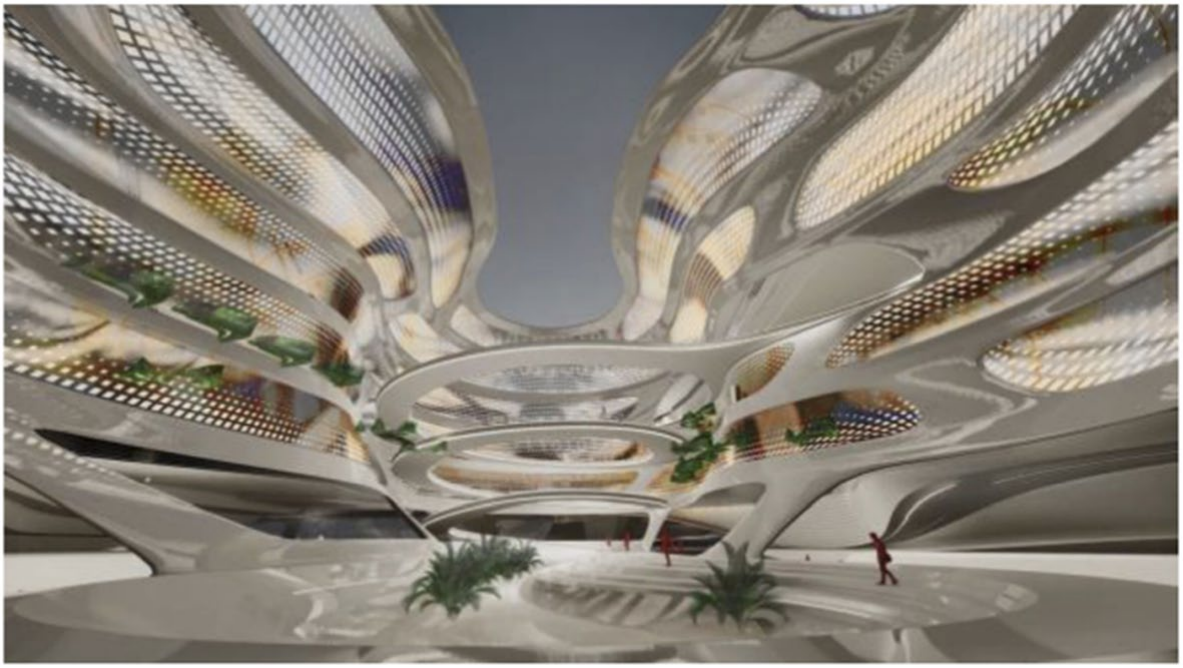
Urban interface of the cyber-urban incubator – façade and courtyard. ‘Cybertecture’ by Stefan Tzon Manousof, Qi Yang, Amin Yassin, Yang Yu; AADRL Studio ‘Cyber-urban Incubator’, tutors: Patrik Schumacher, Pierandrea Angius

It is one of the ambitions of the author to use the increased dramaturgical design opportunities delivered by the emergence of metaverse architecture to also advance the dramaturgical project in the realm of physical architecture. This has been a longstanding theme within the author’s design research agenda, previously pursuit under labels like ‘responsive environments’ and ‘spontaneous environments’ (Schumacher, 2021). Here is also an important role for AI to enter and empower architectural spaces, both virtual and physical. The ambition is to design an environment where many (if not all) architectural elements become self-directed, learning architectural agents that form a productive collaborative ecology together with the human participants, eschewing remote control for the free unfolding of artificial architectural intelligence. In both the physical and virtual spaces ai-empowered creatures will, more and more, become our valued collaborators. The life-process of the future will thus become a man–machine ecology, in both the physical and virtual spaces that together form the mixed reality our social lives will inhabit (Figs. 10, 11 and 12).

## The liberland metaverse

The Liberland Metaverse is one of several metaverse projects the author is contributing to via Zaha Hadid Architects. Of these projects the Liberland Metaverse has been developed the furthest, and can thus best serve as illustration and occasion to discuss the general thrust of the author’s metaverse ideas and ambitions. This project has reached the stage of an indicative concept design plus an inhabitable ‘proof of concept’ virtual space, open 24/7 to be experienced by invited guests like potential investors. An initial soft launch was held at the occasion of the 7^th^ anniversary of the founding of Liberland for 100 guests with a live stream of the simultaneous Liberland conference broadcast into the primary space of the metaverse. Eight further sound zones, equipped with video screens and screen sharing facility, have been distributed in the virtual incubator building, allowing for various thematic gatherings to take place during the five hour event.

The Liberland Metaverse is an entrepreneurial collaboration (joint venture) of four organisations: Liberland (client), Zaha Hadid Architects (design), Mytaverse (technology) and ArchAgenda (management). Liberland itself is a political enterprise with the intention to establish a libertarian micronation on a seven square kilometre small, uninhabited, disputed piece of land on the Danube, between Croatia and Serbia. The primary motivation behind Liberland – with both its physical and virtual ambitions—is the realisation that the prosperity potentials of our computationally empowered technological civilisation are paralyzed and squandered by a pervasive lack of entrepreneurial freedom, a freedom that needs to restored or expanded. Innovation must be permissionless. The explosion of entrepreneurial and technological creativity in the crypto-ecosystem demonstrates what would be possible if the degrees of freedom that still persist there would be generalized. To revolutionize the mature societies takes too long. We want and need more freedom now, and this can only be achieved by starting fresh with a coalition of enthusiasts and without infringing on incumbent interests. A crypto-metaverse is a viable avenue for this vision, by itself, in its own right, and as a trailblazing anticipation of a physically co-located creative synergy community.

**Fig. 13:**
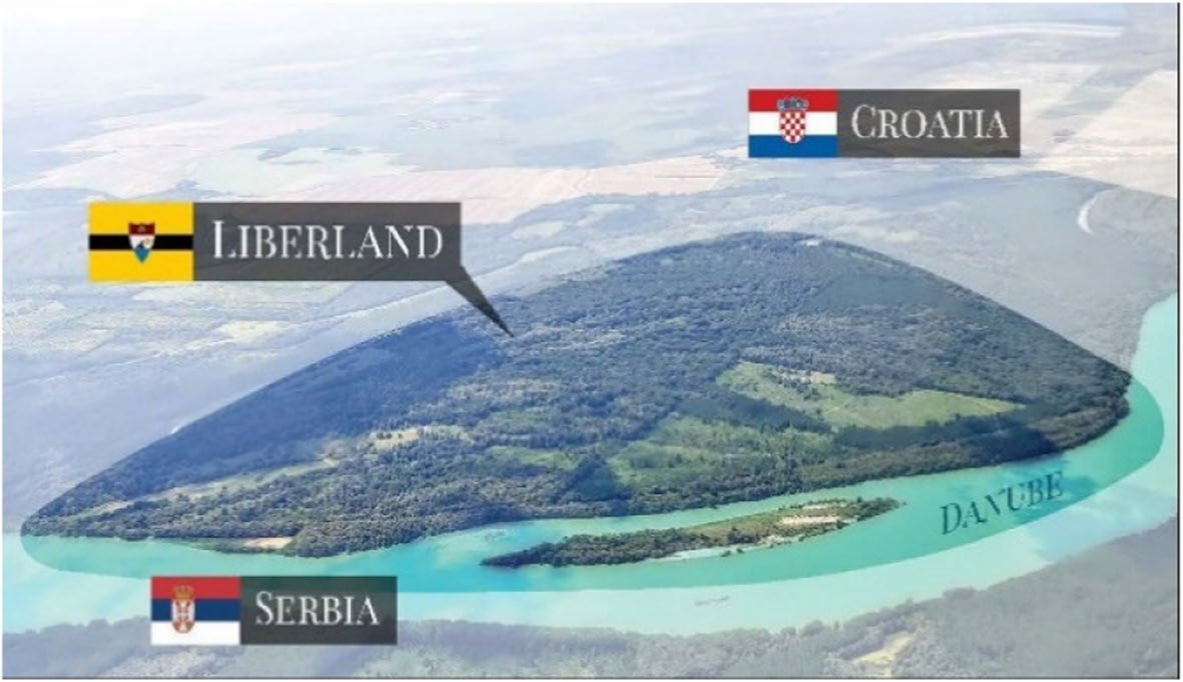
Physical Liberland between Croatia and Serbia

**Fig. 14:**
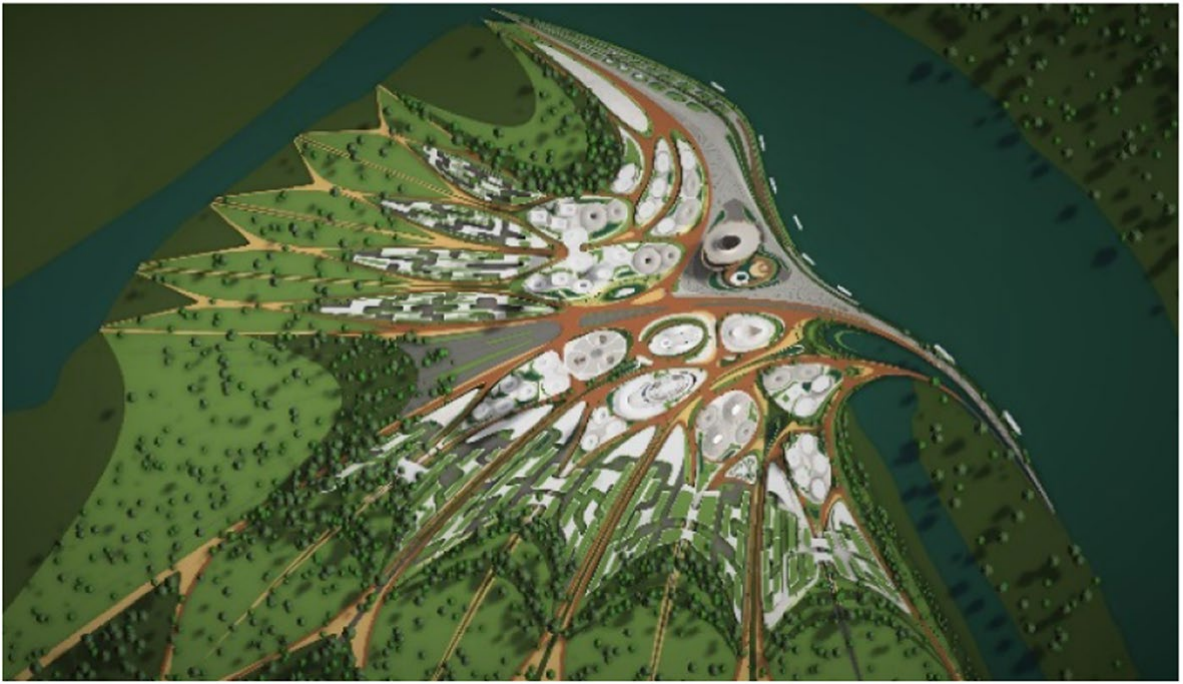
Matching Masterplan for Liberland Metaverse

**Fig. 15:**
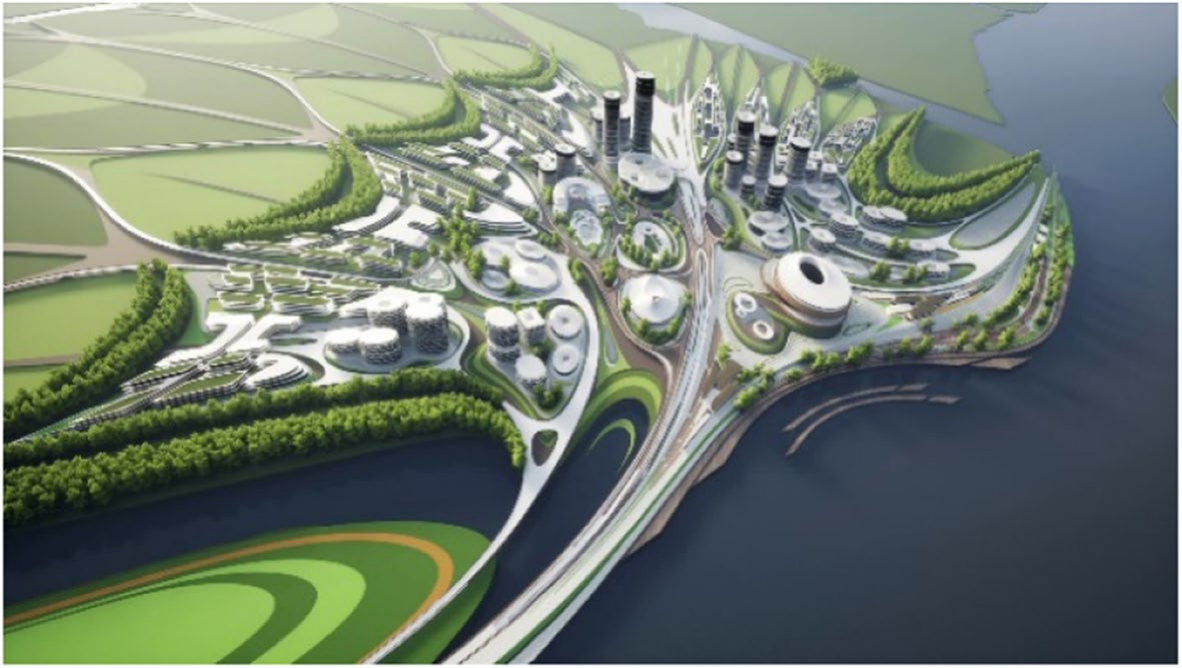
Liberland Metaverse, curated urban core

**Fig. 16:**
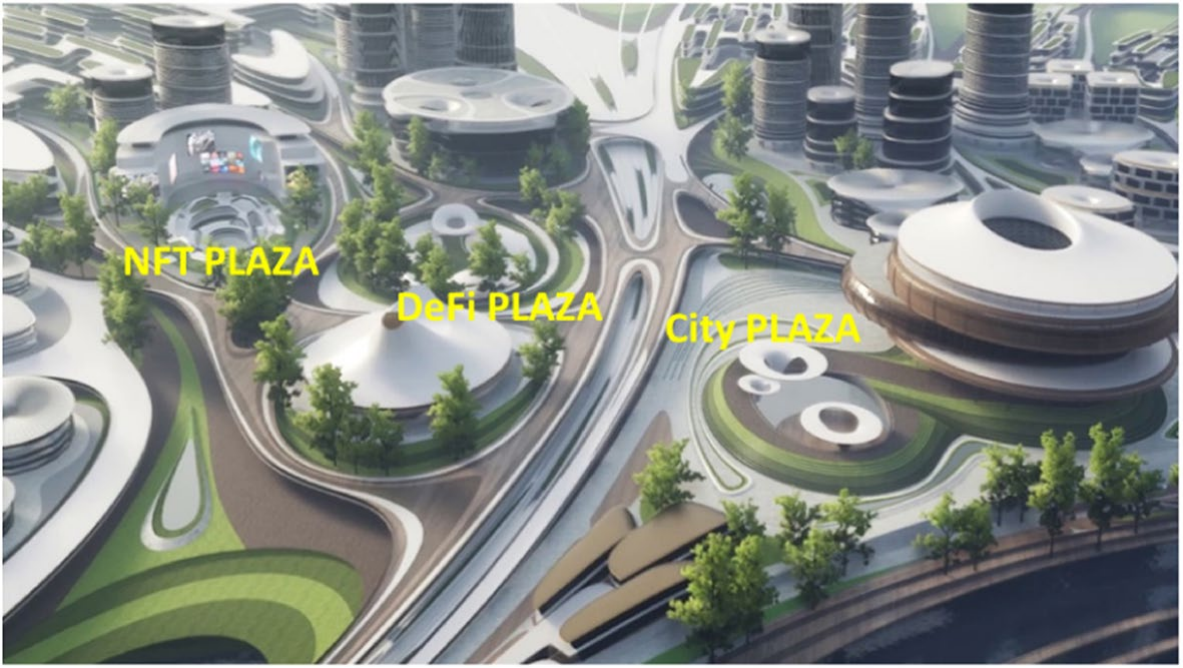
Liberland Metaverse, arrival zone, public spaces

Liberland is well known in crypto circles and has built up a following of would-be citizens of over 600,000. This gives the Liberland metaverse a head-start with respect to achieving its ambitions, namely to become a key site for communication within the crypto ecosystem (Figs. 13, 14,15 and 16).

While the Liberland Metaverse is meant to spearhead the development of Liberland as libertarian micro-nation it will also function as free standing virtual reality realm with the ambition to become the go-to-site for networking and collaboration within the burgeoning Web 3.0 industry, i.e. the metaverse for metaverse developers and the crypto ecosystem at large. The two communities—the community of libertarians who understand that the technology powered prosperity potentials of our times require a revolution in the degree of entrepreneurial freedom and the community of blockchain empowered Web 3.0 developers who realise that their amazing innovative flourishing depends on a permission-less realm of interchange – are congenial and overlap to a significant degree.

The Liberland Metaverse draws inspiration from metaverses like ‘Decentraland’ and ‘Somnium Space’, and from playful social VR environments like VRChat. Like these inspirations, the Liberland Metaverse is inhabited via avatars and allows for virtual land ownership, virtual construction, and (mostly crypto-mediated) economic transactions. However, there are also distinctive differences that strongly differentiate the Liberland Metaverse from ventures like Decentraland. The Liberland Metaverse is meant to operate more like the cyber-urban incubators discussed above. It is not a free-for-all playing field but a designed high-performance work environment for an industry-specific cluster of creative start-up companies. With its focus on the crypto ecosystem the Liberland Metaverse is a quite distinct niche metaverse. This distinguishes it from more generic projects (like Decentraland) where anything goes, including online gambling districts. What is also distinctive is that the Liberland Metaverse embraces realism and sees itself as continuous with productive social reality, and distances itself from the world of video-gaming that still dominates most current metaverse projects. The Liberland Metaverse becomes part of societal reality and an integral part of social production and societal reproduction. This conception of the metaverse does not offer an alternate reality or second life or any escape from social reality or societal life but instead enhances the life of society. To clearly see this concept of a seamless unity of social reality envisioned here it might be helpful to refer to Niklas Luhmann’s conception of society as consisting of nothing but communications, understanding as the totality of communications (Luhmann, 2012/2013). This conception of the metaverse contrasts with the many current “metaverses” that—like Sandbox, Roblox, Axie Infinity, Netvrk, Wilder World etc.—which are in fact primarily multi-user computer games, with added in-game economic transactions. This conception of a reality-based rather than fiction-based metaverse gives a concrete meaning to what David Chalmers calls the Reality Question, namely “are virtual worlds real or illusory?” (Chalmers, 2022, p.9). Chalmers question remains a vague, free-floating philosophical question. This leaves his affirmative answer that virtual worlds/objects are as real as the physical world and its objects (Chalmers, 2022, p.14) equally free floating. Chalmers’ assertion that virtual and physical places are “equally real” (Chalmers, 2022, p.14) is running idle as philosophical truth claim but is a welcome thesis here when filled with concrete meaning. This thesis is here being interpreted and operationalized here via the practically significant distinction between two types of virtual worlds, those which—like the Liberland Metaverse—are real in the sense of partaking and continuing global societal reality (world society) and those which are unreal in the sense of not being intent on this continuation. The real metaverse is framing societal communicative interaction. The unreal metaverse is part of the entertainment industry and has a rather different societal function. In Luhmann’s sociology its probably located either, like movies, in the function system of the mass media, contributing by constituting a globally shared world view, or in the art system, contributing by sensually confronting our social reality with alternative possibilities. This distinction between real and unreal metaverses implies no value judgement. Both architectural framings and entertaining fictions can be regressive or progressive. The point of the distinction here is to clarify the reality of the Liberland Metaverse and to note that only this ‘real’ type of metaverse can be a task domain for architecture.

To be sure, the gaming industry has developed all the amazing digital technologies that are now ready to be emancipated and transferred to more expansive tasks. The computer game market is big, but it is only a small niche compared to the generalized concept of virtual interaction spaces that will now invite and frame all domains of human interaction: knowledge exchange, professional collaboration, cultural communication, art, education, political engagement etc.

The architectural and urban paradigm that is most congenial to this idea of a differentiated, evolving, multi-author urban field is: Parametricism (Schumacher, 2009). We therefore predict that the development of the metaverse will boost parametricism. This will also feed back into physical architecture because most organisations and clients will have both physical and virtual venues.

The two primary aesthetic problems we are trying to solve with our urban and architectural designs for the Liberland metaverse can be stated as follows:

- 1. On the urban scale: To maintain a global sense of unity and identity while accommodating a rich diversity of districts, institutions and congenial architectures.
- 2. On the architectural scale: To maintain legibility in the face of complexity. The task is to maintain inter-visibility and inter-awareness in mixed use scenarios with very complex spatial arrangements. This is achieved by the use of fluidity and spatial porosity, allowing for deep, layered vistas.

**Fig. 17:**
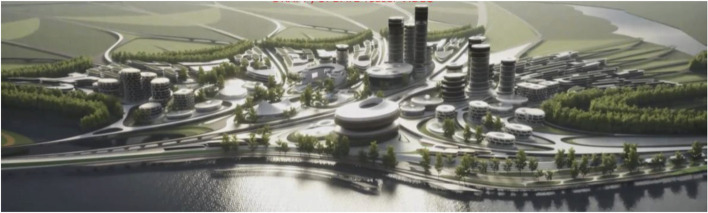
Liberland Metaverse, City hall facing Danube

**Fig. 18:**
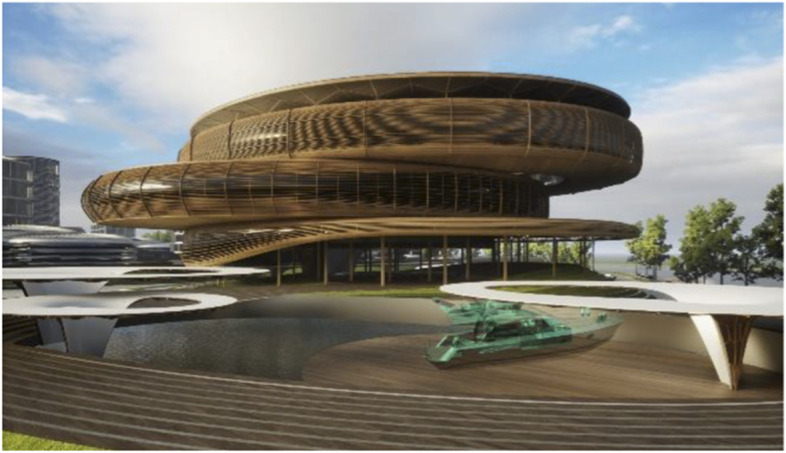
Liberland Metaverse, City Hall exterior

**Fig. 19:**
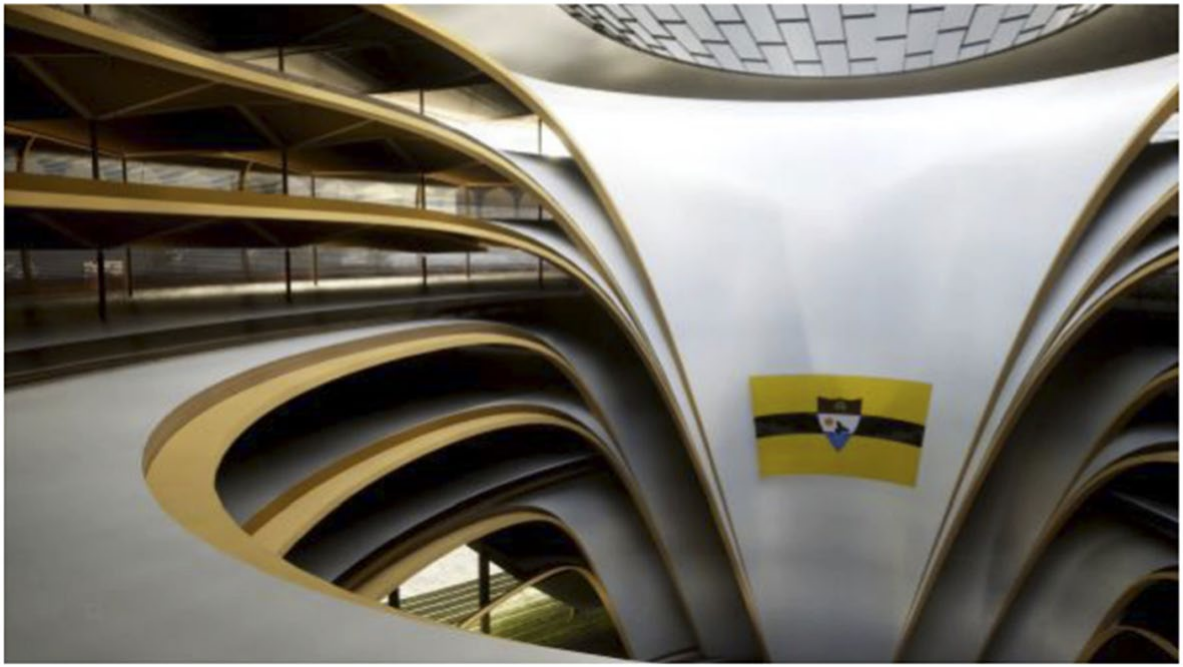
Liberland Metaverse, City Hall Interior

**Fig. 20:**
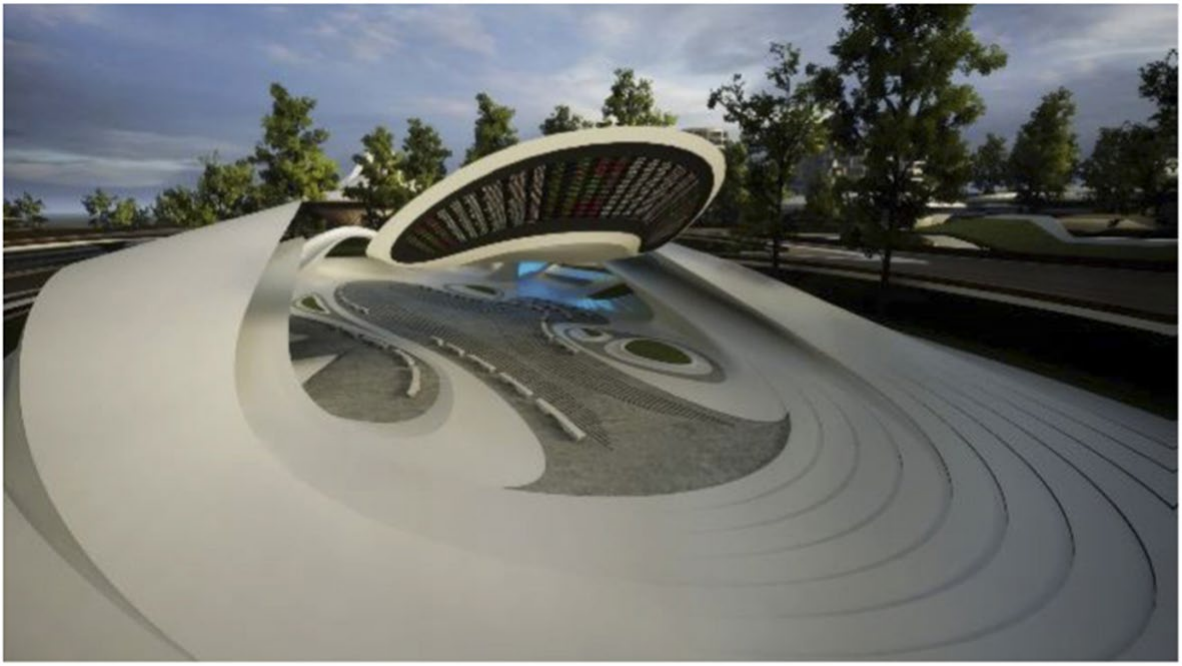
Liberland Metaverse, DeFi Plaza

**Fig. 21:**
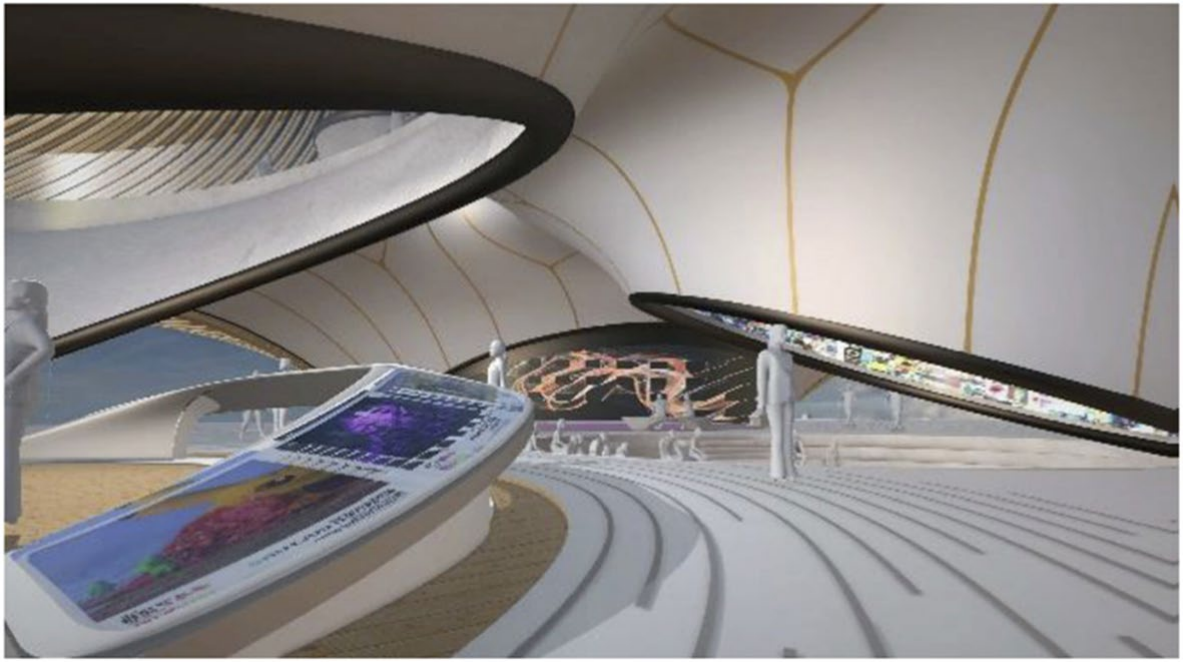
Liberland Metaverse, NFT Art Gallery, interior

**Fig. 22:**
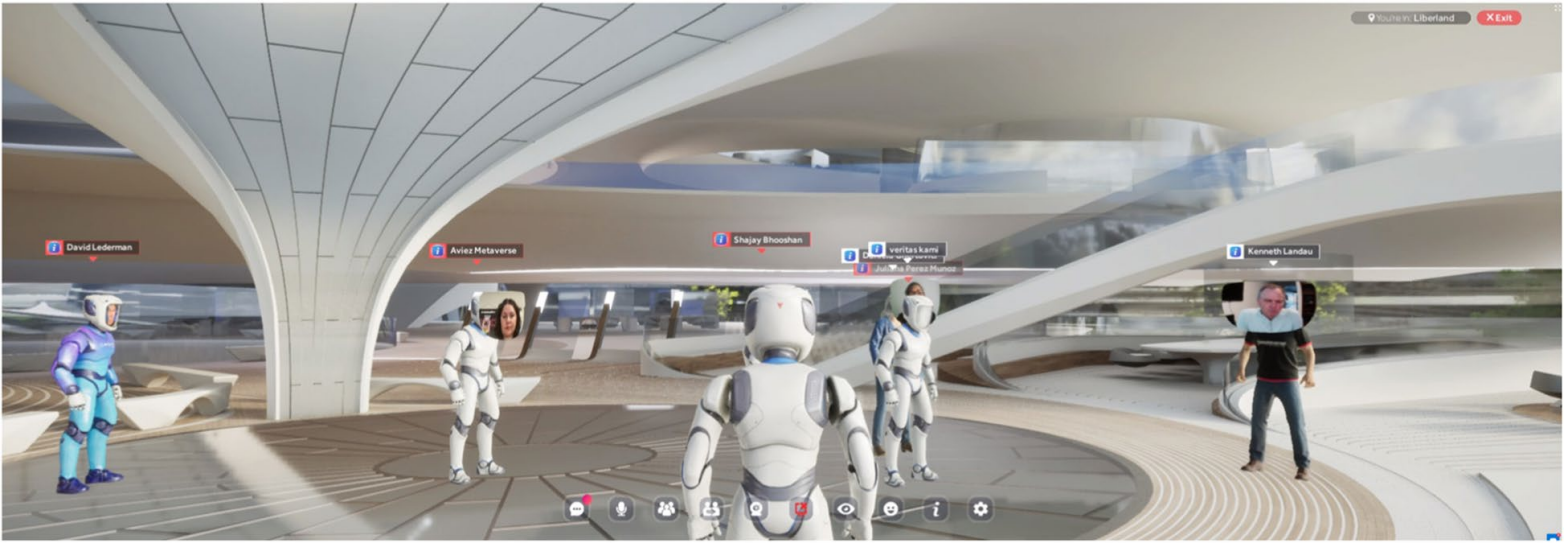
Liberland Metaverse, Multi-tenant Crypto-incubator building, interior – functional proof-of-concept

**Fig. 23:**
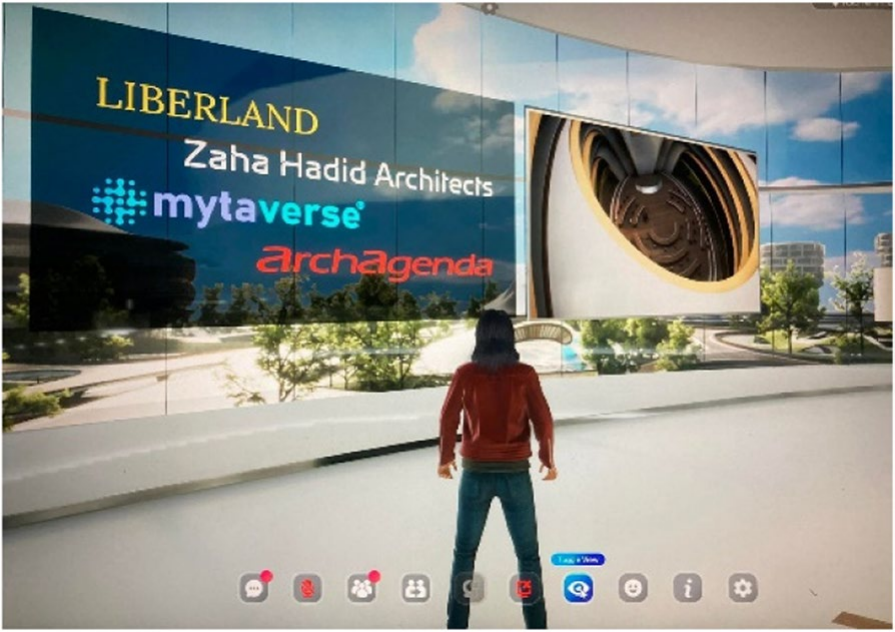
Liberland Metaverse, Snapshots from 7th Anniversary of Liberland, celebrated inside the Incubator

**Fig. 24:**
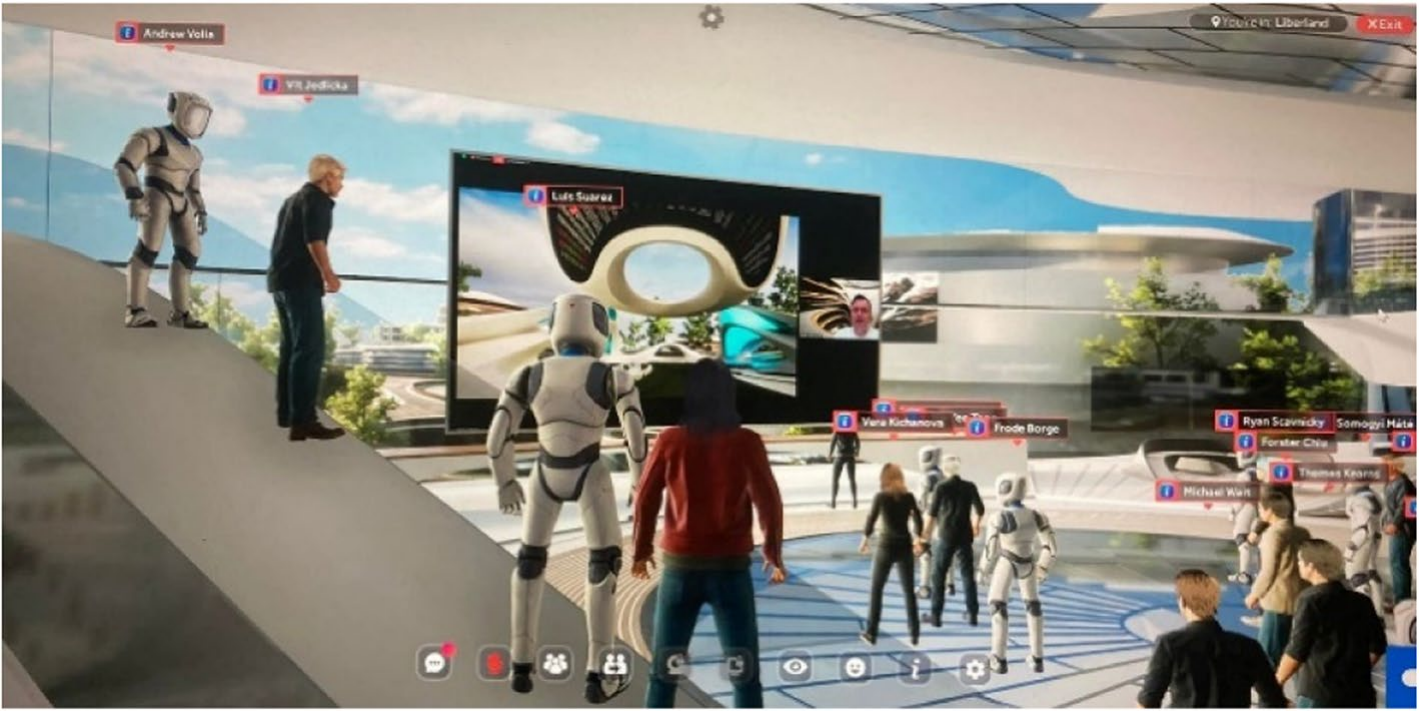
Liberland Metaverse, Snapshots from 7th Anniversary of Liberland, celebrated inside the Incubator

As long as we have physical bodies we’ll need physical environments. As elaborated above, virtual environments are as real as physical environments and social reality exists and continues seamlessly across this divide. Virtual and physical environments are ideally designed together. The key advantages of virtual environments are their global accessibility and their adaptive, parametric malleability. As elaborated in the preceding chapter, we can foresee the interweaving of virtual and physical spaces. This is also anticipated in our conception of the Liberland metaverse. Its land is a one-to-one digital twin of the physical land mass of Liberland (beautifully situated at the Danube river between Serbia and Croatia). Our conception of the metaverse is based on realistic design and photo-realistic rendering. We believe this, at least in the initial stages of metaverse development, allows for the fullest exploitation of the city analogy, utilising our innate and learned intuitive cognitive capacities with respect to orientation, wayfinding and the reading of subtle aesthetic social atmospheres and situations. This realism in our cyber-urban conception also allows for the later physical realisation of the designed metaverse spaces in the physical Liberland, to any desired extent (Figs. 17, 18, 19, 20, 21, 22, 23 and 24).

Two powerful innovations are coming together in the metaverse we envision with Liberland: First the immersive internet allowing for a new level of life-like spontaneity in social interactions, and second the internet of value allowing for truly global economic collaboration without gate keepers, no matter which passport participants hold. We conceive of the metaverse as an open platform, based on freely circulating open source insights and technologies, building on and participating in the culture of permission-less innovation that has fuelled the crypto ecosystem in recent years. As Vitalik Buterin has insightfully argued, the most crucial currency for crypto platform projects is legitimacy within the wider ecosystem and community of participants (Buterin, 2021). That is why we believe that the open, decentralised, community owned versions of the metaverse—organised as transparent DAOs—will win out over centralised corporate ventures. This fits into an emerging world where the end to stagnation and a boost to prosperity for all requires that centralised power blocks give way to global markets and discourses. The metaverse will in turn accelerate this larger transformation.

**Fig. 25:**
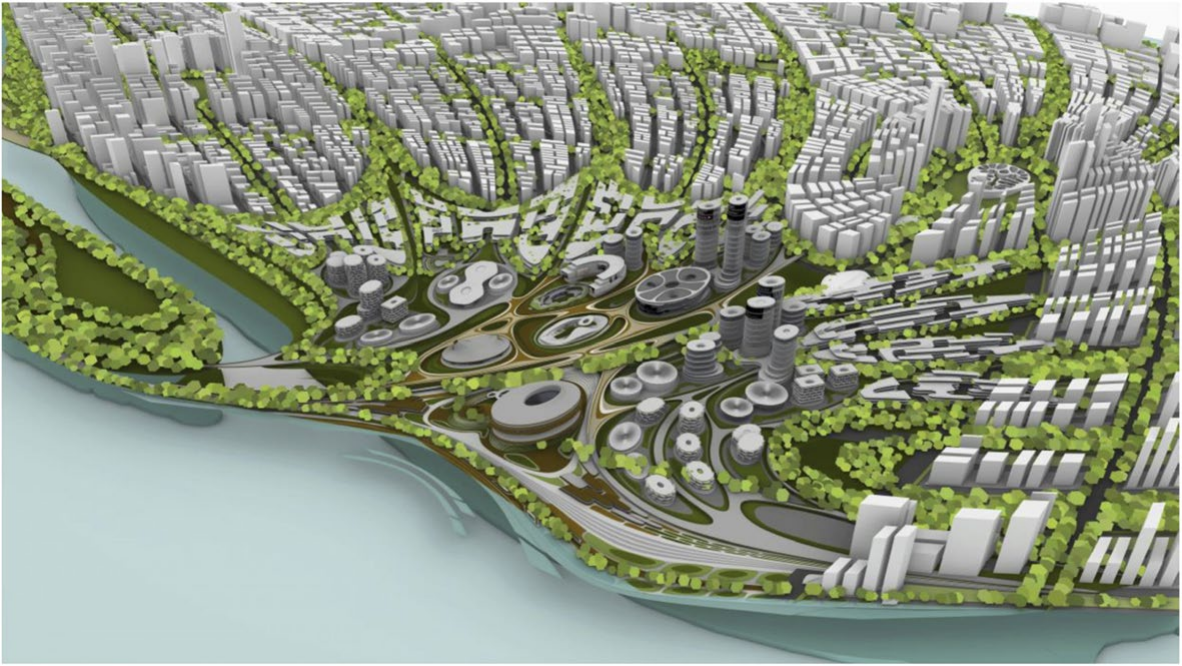
Liberland Metaverse, abstract visualisation of virtual city fabric on approximately 2000 land parcels

**Fig. 26:**
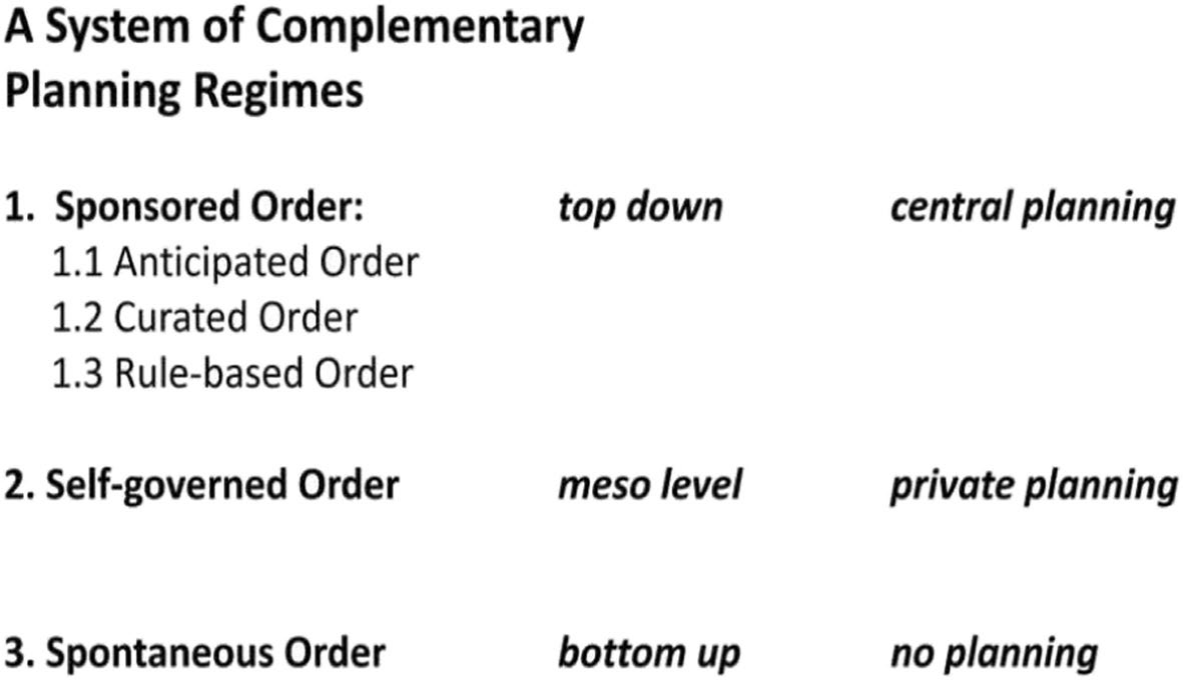
Liberland Metaverse planning zones: sponsored urban core, self-governed districts, spontaneous free-zone

**Fig. 27:**
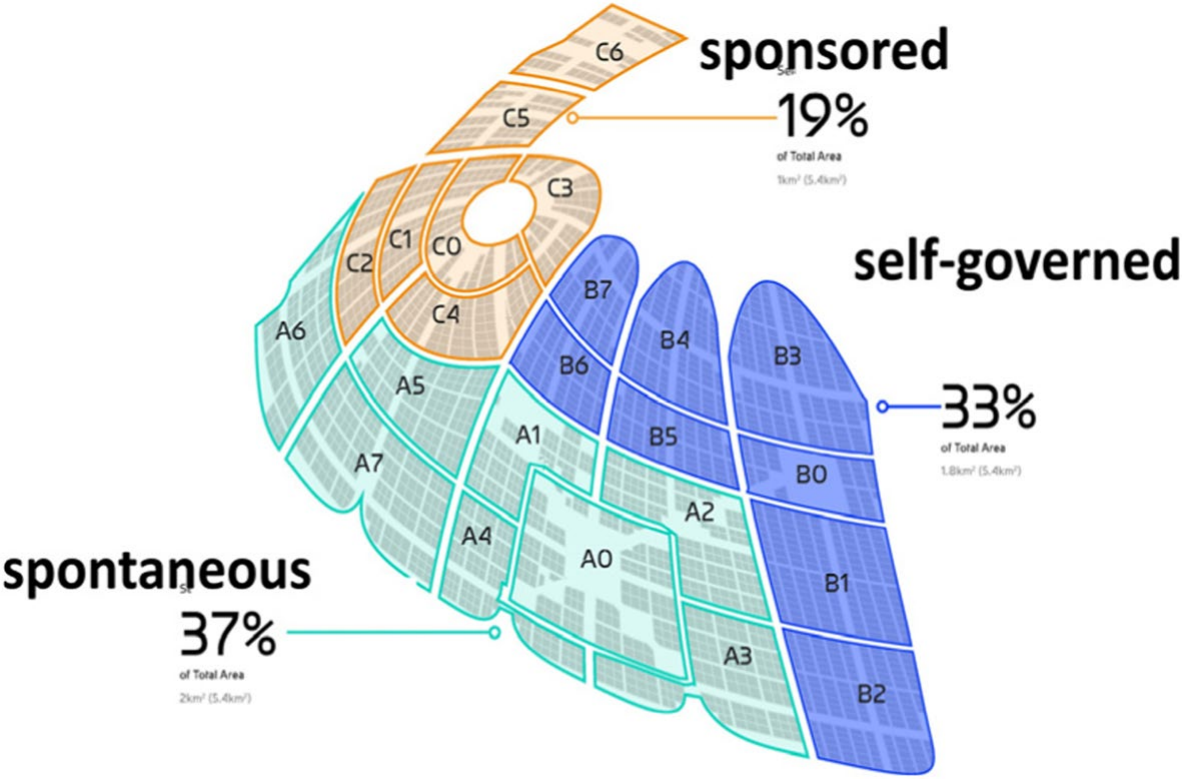
Liberland Metaverse planning zones: sponsored urban core, self-governed districts, spontaneous free-zone

In Liberland parcels of land will be sold with covenants in accordance with a nested system of different planning regimes: a central curated urban core, surrounded by a layer of districts where we encourage urban self-governance, and finally zones where the absence of urban planning allows for a spontaneous order via a free-wheeling discovery process. The key idea here is thus the urban meta-policy of a simultaneously plurality of approaches rather than a single one-fits-all planning regime (Schumacher, 2020). The strategic decision would thus be to offer choice to potential developers, investors, buyers and end users. In the overarching context of government as contractually bound service and the pursuit of governance innovation, it is important to experiment and use the initial market feedback from potential investors and then from the measured market performance of built results, for a pragmatic discovery and selection process concerning urban development regimes and policies. Policy regimes aim at an urban order implying an optimal co-location pattern that maximises positive externalities, i.e. functional spill-overs or synergies, and minimises negative externalities. The maintenance of a nimble, flexible openness to future opportunities is another concern to be considered (Fig. 25, 26 and 27).

The planning regimes introduced here apply to both metaverse urbanism and physical urban development. The assumption here is that the various planning regimes listed above are distributed across the land available to Liberland, initially virtual and later physical, so that each regime is instantiated and tested in at least one zone or urban district. Regimes can then be expanded or contracted, first based on uptake during the marketing and land sale phase, and later in accordance with the actual urban development experiences on the ground.

## Theses on the advent of the metaverse

This paper presents reflections and speculative anticipations of the coming era of the metaverse, not only as opportunity for entrepreneurs and architects, but also as exiting, progressive development for society at large, culturally, economically and politically. The following 12 theses on the type of metaverse described and promoted here may serve as summary and conclusion, as well as a manifesto for work to come:

### Thesis 1: total transformation

The metaverse will deliver vivid tele-presence, co-location synergies, explorative browsing, immersiveness, collective experiences etc. The uptake of this opportunity will be universal.

All websites will spatialise, all organisations will move into the metaverse, all physical venues will be augmented or substituted by functionally equivalent virtual venues.

### Thesis 2: a single reality

The metaverse is neither a game, nor fiction. Virtual reality in the metaverse will be no less real than the physical reality in our cities. Physically and virtually mediated social communicative interactions are equally significant and together form an undivided continuous social reality. There will be both competition and cooperation within and across these realms.

### Thesis 3: cyber-urban fusion

Cyberspace will fuse with urban space implying a radical transformation of built architecture and urban life. Urban and architectural spaces become interfaces and windows into the virtual world. Mixed reality—mixing physical and virtual co-presence -will be pervasive.

### Thesis 4: global meta-metropolis

There will be many metaverses but the factors that promote big world cities—agglomeration economies (service efficiencies & servicing long tails) & co-location synergies in terms of collaborative networks – will also drive concentration in large metaverse platforms.

### Thesis 5: distributed ownership

Like a city, a metaverse is a shared platform. It will flourish when it is globally accessible, open source, permissionless. This implies a continuously evolving diverse self-organising community of interest, or community of the invested. This is more compatible with the decentralised forms of governance explored within the crypto ecosystem than with corporate proprietorship.

### Thesis 6: metaverse urban planning

The strategic meta-policy: Start with a simultaneous plurality of approaches, i.e. to work with several (district-based) planning regimes rather than with a single blanket regime, thereby initiating a comparative experiment and discovery process. The initial market feedback investors, and later the end-user market success of the built results, will guide the selection and differential expansion/contraction process with respect to urban development regimes.

### Thesis 7: the architect’s take-over

In the coming age of VR empowered cyberspace it will be architects and no longer graphic designers who will design the coming 3D immersive internet: the metaverse.

### Thesis 8: architecture’s essence distilled

This expansion of architecture’s remit will further distil the discipline’s essence and core competency, namely the spatio-visual ordering of communicative interaction, upgraded via investment into the subdisciplines of spatiology, phenomenology, semiology and dramaturgy.

### Thesis 9: switching technological constraints

While the expansion of the architectural discipline into the virtual realm is seamless and leaves its essential expertise and values untouched, no of the supporting/constraining engineering disciplines is joining this expansion. There is a total substitution of the engineering stack. New collaborators are now imposing new constraints: polygon budgets and transmission bandwidth etc.

### Thesis 10: imperative realism

The metaverse exploits the analogy of the city, utilising our ability of navigating urban and architectural spaces as well as our ability to recognize places and social situations. This requires a high degree of realism in terms of plausible design and photo-realistic rendering because the semantics attaches to atmospheric values that might not survive abstraction.

### Thesis 11: evolving beyond realism

While a close adherence to the analogy with urban architecture is important to begin with, we can expect a gradual emancipation and evolution of metaverse native forms of semiological articulation, navigation and interaction, exploiting the inherently different freedoms and constraints of the medium. However, the expected cyber-urban fusion will always insure a tie back to the physically instantiated urban and architectural semiology.

### Thesis 12: Congenial Parametricism

As native digital style Parametricism is congenial with the ambitions of the metaverse and will become the preferred style here. This will feed back into architecture at large and accelerate the dissemination of parametricism. The advantages of a high density, complex, variegated, legible spatio-morphological order and the requirement for continuous adaptation to changing contexts and interaction scenarios, persist in the metaverse and can only be delivered by parametricism.

## Data Availability

Not applicable.
